# Magnetic Resonance Imaging of the Knee in the Presence of Bridging External Fixation: A Comparative Experimental Evaluation of Four External Fixators, Including Dolphix^®^

**DOI:** 10.3390/jfmk7010004

**Published:** 2021-12-30

**Authors:** Francesco Addevico, Alberto Simoncini, Giovanni Solitro, Massimo Max Morandi

**Affiliations:** 1 Department of Orthopedic and Traumatology, Niguarda Hospital, 20162 Milano, Italy; Francescoaddevico@gmail.com; 2 Department of Radiology, Louisiana State University Health-Shreveport, Shreveport, LA 71103, USA; albertosimoncini@yahoo.com; 3 Department of Orthopaedic Surgery, Research Lab, LSU-Health-Shreveport, Shreveport, LA 71103, USA; massimo.max.morandi@gmail.com; 4 Department of Orthopaedic Surgery, University of Florida, Jacksonville, FL 32816, USA

**Keywords:** MRI imaging, temporary bridging external fixator

## Abstract

Performing MR investigation on patients instrumented with external fixators is still controversial. The aim of this study is to evaluate the quality of MR imaging of the knee structures in the presence of bridging external fixators. Different cadaveric lower limbs were instrumented with the MR-conditional external fixators Hofmann III (Stryker, Kalamazoo, MI, USA), Large external Fixator (DePuy Synthes, Raynham, MA, USA), XtraFix (Zymmer, Warsaw, IN, USA) and a newer implant of Ketron Peek CA30 and ERGAL 7075 pins, Dolphix^®^, (Citieffe, Bologna, Italy). The specimens were MR scanned before and after the instrumentation. The images were subjectively judged by a pool of blinded radiologists and then quantitatively evaluated calculating signal intensity, signal to noise and contrast to noise in the five regions of interest. The area of distortion due to the presence of metallic pins was calculated. All the images were considered equally useful for diagnosis with no differences between devices (*p* > 0.05). Only few differences in the quantification of images have been detected between groups while the presence of metallic components was the main limit of the procedure. The mean length of the radius of the area of distortion of the pins were 53.17 ± 8.19 mm, 45.07 ± 4.33 mm, 17 ± 5.4 mm and 37.12 ± 10.17 mm per pins provided by Zimmer, Synthes, Citieffe and Stryker, respectively (*p* = 0.041). The implant of Ketron Peek CA30 and ERGAL 7075 pins showed the smallest distortion area.

## 1. Introduction

Bridging external fixation of the knee must be implemented for the temporary emergent stabilization of the knee joint [1,2]. There are many injuries that can benefit from a temporary surgical stabilization of the knee, including tibial plateau fractures, knee dislocations, and femoral condylar fractures [2] External fixation plays a role in orthopedics’ traumatology as an optimal management while awaiting the healing of soft tissue or the right timing for definitive surgery [2,3,4,5]. The concomitant utilization of magnetic resonance imaging (MRI) is an indispensable diagnostic tool in several orthopedic trauma scenarios and has widely increased in the last decade [6,7,8]. However, performing an MRI on patients instrumented with temporary external fixators is still controversial and subject to debate [1].

MRI is widely considered an indispensable diagnostic tool in several scenarios secondary to acute trauma, as polytrauma patients often require MRI after resuscitation [1]. In case of an injury involving the knee, several authors reported that the implementation of an MRI diagnostic examination could be useful in elaborating on the diagnosis and more precise surgical planning [9,10,11]. MRI has been associated with a more accurate classification of fractures involving the knee, a better identification of occult fracture lines and more detailed measurement of displacement of bone fragments in tibial plateau fractures compared to plain radiographs or computed tomography [12,13]. Another aspect specifically related to the use of MRI is the assessment of soft tissues such as cruciate and collateral ligaments after knee dislocation, where the use of temporary stabilization is often advocated [6]. Gillig J.D. et al. reported that MRI imaging in Schatzker IV, V and VI tibial plateau fractures better visualized bone landmarks and could avoid the use of computer tomography (CT), experiencing no complications [14].

Typically, in the case of an acute, displaced tibial plateau fracture, application of a bridging external fixator across tibia and femur is indicated. Once the ligamentotaxis has been obtained, the subsequent step involves computerized axial tomography (CAT scan) to assess the damage and plan for surgical definitive fixation. In daily practice, because of the nature of the injury combining bony and soft tissue alterations, orthopedic surgeons should request more often MRI scans of the knee following bridging temporary external fixation of the joint [14,15].

The greatest concerns of MRI utilization on patients with external fixator continue to be the potential alleged injuries due to the loosening or migration of components [16], implant heating leading to thermal lesion [17], and signal distortion with interference in images interpretation [18,19]. All of these hazards have been related to the presence of ferromagnetism in the components of the stainless-steel material; for example, delta ferrite in the rods or clamps [7,20]. The magnetic field has the potential to cause movement or dislodgement, induction of electric current, and/or excessive heating of metallic materials [8,21,22]. The new generation of devices has seen a large elimination of delta ferrite, as they are now primarily composed of composite materials, titanium, aluminum [1], carbonium and Kevlar with non-ferromagnetic and non-conductive properties. A prime example is the Dolphix^®^ (Citieffe, Bologna, Italy), made of Ketron Peek CA30, a semi-crystalline polymer based on polyetheretherketone resin loaded with 30% carbon fibers reinforcement. An 11 mm diameter ERGAL 7075 aluminum-alloy pin clamp cylinder is the sole metal part in the clamp, fixed to the bone with standard titanium pins.

In 2014, the FDA classified external fixators with four different labels [23,24]. Devices nonmetallic, nonconducting and nonmagnetic are defined “MRI safe”, even if it is underlined that quality of images could be affected by the presence of the devices. “MRI compatible” are those devices defined as not affecting the quality of diagnostic information and MRI safe. “MRI conditional” indicates those devices that have been set up with predetermined environment specifications that should be respected to avoid hazards [25]. Finally, devices labeled “MRI unsafe “are hazards in all MRI environments. By definition, MRI safe medical devices do not contain any metal and are composed entirely of materials that are electrically nonconductive, nonmetallic, and nonmagnetic. An MRI safe medical device can safely be taken into any MR environment. The MRI safety information for an MRI unsafe medical device should indicate that the medical device is MRI unsafe and should remain outside the MRI scanner room.

Despite this classification, radiology departments considered restricting the use of MRI in the management of trauma patients implanted with external fixators for medical and legal reasons [14]. According to the orthopedic physician requiring the MRI, the final decision to perform or not perform the exam is usually ultimately made by a radiologist or a radiology technician, balancing on diagnostic benefit and potential risks of harmful effects [26]. In fact, in the past, clinical reliability of the MRI images has been debated. The FDA labeling addresses the safety of the procedure but does not provide indication on the clinical reliability of the MRI obtained images [12,27]. More recently, Elsissy et al. found that even MRI-compatible external fixators reduce artifact noise from the images, but do not completely eliminate the noise [20].

Therefore, in this study we aim to assess the clinical validity of MRI images obtained for the evaluation of knee soft-tissue structures. More specifically, we target evaluation of different MRI conditional labeled external fixators in terms of MRI signal and clinical validity. Furthermore, we evaluated the areas of distortion that have been shown due to the presence of pins.

We hypothesize that the use of labeled MRI conditional external fixators in cases of knee spanning external stabilization is clinically valid regardless of the brand, while differences between those are related to the area of distortion due to the presence of pins and, ultimately, to the physical components of the implants. The Dolphix^®^, made of PEEK CA30 material and ERGAL 7075 aluminum alloy pin clamp, is an implant that appears extremely safe and effective.

## 2. Materials and Methods

### 2.1. Specimens

The study was performed on cadaveric lower limbs instrumented with four different brands of external fixators available at our institution. The fresh frozen lower limbs, harvested from cadavers with age of 79.5 ± 6.3 yrs, were thawed 48 h before instrumentation and imaging at room temperature that was measured to be 19.7 °C. A CT study was performed on the samples to rule out the presence of metallic devices previously implanted.

### 2.2. Surgical Technique

An orthopedic trauma surgeon implanted the devices, as they had to bridge an intercondylar femur fracture without extension to the femur shaft. Pins were implanted at the first instrumentation of limbs by skin incision, drilling of the first cortical bone with power-drill of 4 mm diameter and the pins then screwed in by hand reaching the second cortical bone. Each limb was instrumented for the first time with a different ex fix that was then placed after the imaging on the subsequent limb so that sequences of the implant could not be considered a bias. Pins were placed in the same holes and at the same depth to recreate the same configurations.

### 2.3. External Fixator Configuration

**Figure 1:**
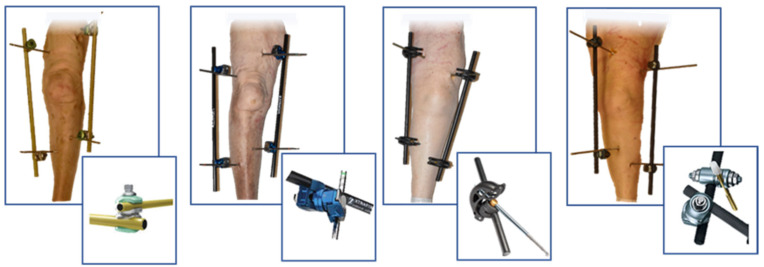
Example of the legs instrumented with the four brands of external fixators by “H” configuration. Down from left are reported samples of the devices of Hofmann III^®^, XtraFix^®^, Dolphix^®^ and Large E.F^®^.

The MRI conditional external fixators considered were: Dolphix^®^, (Citieffe, Bologna, Italy), Hofmann III (Stryker, Kalamazoo, MI, USA), Large external Fixator (DePuy Synthes, Raynham, MA, USA) and XtraFix (Zymmer, Warsaw, IN, USA). They were all instrumented to recreate an “H” construct as shown in Figure 1. Such construct was obtained by placing two pins in the femur, one medially and one laterally superior to the patella and two pins placed, one medially and one laterally inferior to the tibial tubercle. The medial and lateral pins were connected by two 300 mm bars linked themselves by a transverse bar [28,29]. The bars utilized by Stryker, DePuy and Zimmer were 11 mm of diameter, while Citieffe’s were 12 mm. The pins of the different brands were all self-drilling, 200 mm of length while in diameter of 6mm for the femur and of 5 mm for the tibia. The Citieffe and DePuy pins were in titanium while the Stryker and Zimmer were in stainless steel.

### 2.4. Magnetic Resonance

MRI scans of the specimens were performed before and after instrumentation, in order to use the images of the un-instrumented leg as control. The imaging was performed using a general GE Signa at 1.5 Tesla (GE Healthcare, Chicago, IL, USA) with the sequence TR: 2700–2980 ms and TE: 16.6–16.7 ms with an 18 cm cardiac rec. coil.

### 2.5. Clinical Validity

The MRI scans were qualitatively evaluated by a pool of radiologists from the institute who were asked to declare if they considered the images diagnostics on a scale from 1 to 5 focusing on menisci, cruciate ligaments, patellar tendon and bone marrow near the joint.

### 2.6. Images Quantification

A quantification of the images was performed calculating signal intensity (SI), signal to noise (SNR) and contrast to noise (CNR) in the five regions of interest (ROI). The SNR was defined as the ratio between the average signal intensity to its standard deviation (SNR = SItissue/SDtissue) while the CNR was measured as the ratio between the average signal intensity in the ROI subtracted from the average signal intensity of the noise to the standard deviation of the background noise signal intensity (CNR = SItissue-SInoise/SDnoise).

A background signal was defined by placing a 600 mm^2^ circular ROI next to the limb in proximity to the tibiofemoral joint at the slice depth of the tibial eminence on coronal proton density (PD) weighted images. Image distortion in each instrumented limb was also evaluated through the length of the area free of visible artifacts (LAFA) measured from the joint line.

The five ROIs were detected on the coronal PD weighted images as: anterior cruciate ligament (ACL = rectangular 20 mm^2^ ROI near the ligament femoral attachment), posterior cruciate ligament (PCL = polygonal ROI just posterior to the PCL femoral attachment on coronal plane), medial meniscus (MM = ROI of 6.3 mm^2^ within the meniscus body on a slice at the depth of the tibial eminences), patellar tendon (PT= a 2 mm × 7 mm (14 mm^2^) rectangular ROI within the patellar tendon slice at its mid-length on coronal plane) and bone marrow (BM = a square 100 mm^2^ ROI at a level 10 mm inferior to a horizontal line connecting the medial and lateral tibial plateaus at the tibial eminence).

In order to compare the influence of the four different devices on the quality of the MRI images, the authors created a mean of each parameter (SI, SNR and CNR) computed in the five different ROIs in each leg instrumented with the different devices. Value of SI, SNR, CNR registered for the fixators in the five different ROIs and measures of LAFA were analyzed using ANOVA to evaluate differences among external fixators, while individual variations from the control group were recorded using paired *t*-test at a level of significance of 0.05 in Microsoft Excel (Microsoft Corporation, Redmond, WA, USA).

### 2.7. Length of Areas Free of Distortion from the Joint

In each instrumented limb it was calculated, using the coronal PD weighted images of the femurs, as the length of radius of the distortion of the pins and the length of the area free of artifacts due to the presence of pins calculating from the joint line. We then ran a mean of these measures, calculating which device had the bigger area free of distortion by ANOVA test.

## 3. Results

### 3.1. Value of the MRI as Diagnostic Tool

The MR images were all declared equally useful for the diagnosis. All the images were scored 5/5 by the radiologist asked to review the images. A meniscal tear was diagnosticated on the Limb B and it was detectable and classifiable in each MR study performed on the limb.

### 3.2. MR Images Quantification

**Figure 2:**
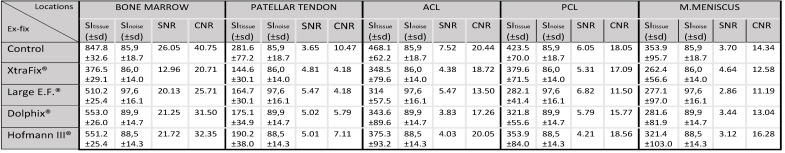
Values of Signal Intensity of tissue (SItissue), Signal Intensity of noise (SInoise), Signal to Noise (SNR), and Contrast to Noise (CNR) in the different regions of interest (ROIS) stratified for devices.

The results of Signal Intensity of the tissue (SI_tissue_) and its standard deviation (SDt), SI of the noise (SI_noise_) and its SD, Signal to Noise (SNR = SI_tissue_/SD_tissue_) and Contrast to Noise (SI_tissue_-SI_tissue_)/SDNoise) are reported in Figure 2.

The average SI_tissue_ in the bone marrow control group was found to be higher than the instrumented legs at 847.8 ± 95.92 (*p* = 0.01), with no difference found among the different fixators (*p* = 0.48, see Figure 2. The external fixators ranged from 553.0 ± 36.17 in the Dolphix (*p* = 0.01) to 376.5 ± 281.97 in the XtraFix (*p* = 0.04).

In the patellar tendon the average SI_tissue_ for the control group was 281.6 ± 91.56 (*p* = 0.01) with only Large External Fixator. showing a difference from the control at 164.7 ± 17.49 (*p* = 0.048).

There were no differences noted between fixators in the PCL S_Itissue_ (*p* = 0.48) or meniscus (*p* = 0.92), but for the ACL, the Dolphix^®^ (343.6 ± 106.60, *p* = 0.048) and XtraFix (348.5 ± 96.39, *p* = 0.03) did show a significant difference, unlike the Hoffmann III (375.3 ± 206.63, *p* = 0.30) and Synthes Large External Fixator (314.0 ± 164.54, *p* = 0.10).

The average SNR in the control group for bone marrow was found to be 26.05 ± 7.26 (*p* = 0.36) (see Figure 2), with only the XtraFix showing a difference (12.96 ± 12.48, *p* = 0.04).

For the patellar tendon control group, the average SNR was 3.65 ± 1.31 (*p* = 0.62), with only the Hoffman III showing a difference at 5.01 ± 1.59 (*p* = 0.040). The three remaining fixators ranged from the XtraFix (4.81 ± 1.39, *p* = 0.25) to Large E.F. (5.47 ± 0.94, *p* = 0.36)

In the ACL control, the SNR average was found to be 7.52 ± 3.93 (*p* = 0.48), with only the Xtrafix (4.38 ± 2.67, *p* = 0.04) showing a difference. The other three fixators ranged from Dolphix^®^ (3.83 ± 3.23, *p* = 0.14) to Large E.F. (5.47 ± 3.63, *p* = 0.06) (Figure 2).

There were no differences noted between fixators in the PCL (*p* = 0.31) or medial meniscus (*p* = 0.43), nor were there any differences between the control and a fixator in either location. At the PCL, the control SNR was 6.05 ± 0.73 (*p* = 0.35) with the fixators ranging from the Hoffmann III (4.21 ± 1.79, *p* = 0.22) to the Large E.F. (6.82 ± 0.50, *p* = 0.25). At the medial meniscus, the control was 3.70 ± 0.83 (*p* = 0.47) and the fixators ranged from Large E.F. (2.86 ± 1.98, *p* = 0.69) to XtraFix (4.64 ± 6.74, *p* = 0.37).

The CNR showed no differences in all five locations (see Figure 2). However, the most variability came in the PCL, where the control average was 18.05 ± 5.41 (*p* = 0.16) with the range being the Hoffmann III (18.56 ± 2.92, *p* = 0.06) to the Large E.F (11.50 ± 2.79, *p* = 0.86).

### 3.3. Statistical Analysis

The ANOVA test ran between all groups in the ROIs of bone marrow and patellar tendon showed significant differences in SI (*p* = 0.014 and *p* = 0.012, respectively). In the first one, Citieffe and Stryker external fixators were the only two devices that did not show significant differences compared to the control group. However, an ANOVA test run between data detected only in the instrumented legs did not show significant differences. In the ROI patellar tendon, the Stryker ex-fix was the only one that did not show significative differences with control group. No differences in SI between the devices were found over the ROIs. No differences in SNR were found between all groups in all ROIs. A tendency to significance was detected in CNR between groups in the ROI patellar tendon (*p* = 0.055) where Citieffe and Stryker were the only two devices that did not show significative differences comparing to the control group. The tendency was confirmed by an ANOVA test run without the control group.

### 3.4. Length of Areas Free of Distortion from the Joint

#### Pins Effect

The pins implanted on femurs were placed at distances from the joint line of 72, 85, 108 and 70 mm in leg A, B, C and D, respectively.

The mean length of the radius of the area of distortion of the pins were 53.17 ± 8.19 mm, 45.07 ± 4.33 mm, 17 ± 5.4 mm and 37.12 ± 10.17 mm per pins provided by Zimmer, Synthes, Citieffe and Stryker, respectively (ANOVA test *p* = 0.041).

**Figure 3:**
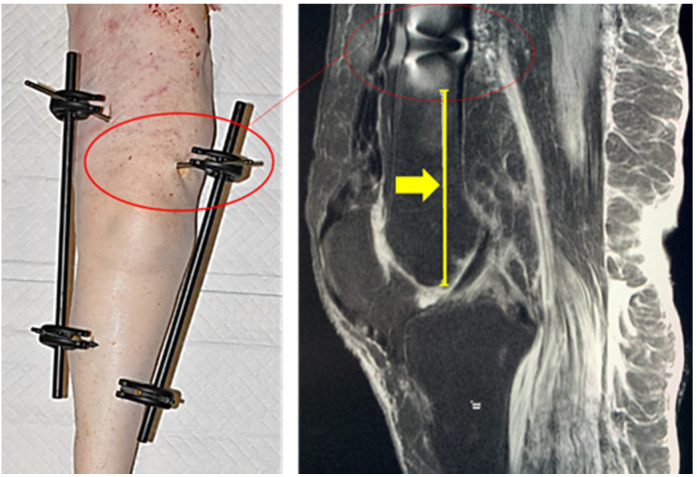
Images of knee (on **left**) implanted with external fixator (Dolphix^@^) and the effect of pin underlined by circle on MRI images (on **right**). The arrow indicates the light of area free of distortion from the pin to the joint line.

The length of the area free of distortion due to the presence of pins of each leg calculating from the joint lines were as described in Figure 3.

**Figure 4:**
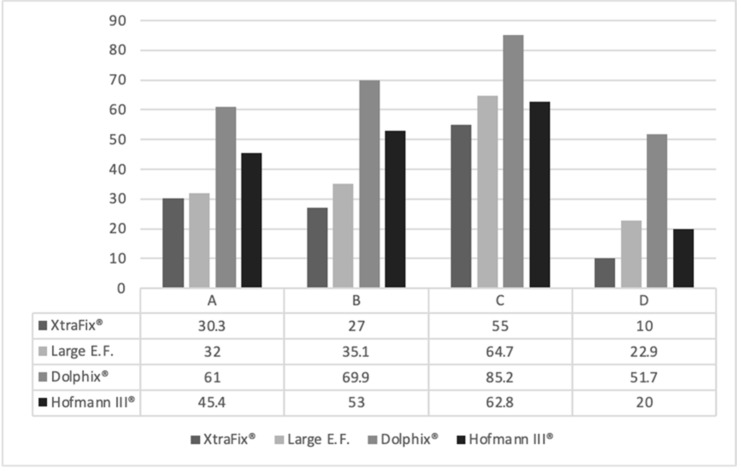
Plot describing the length of the areas free of distortion in mm calculating from the joint line in the four legs (A, B, C and D) instrumented with the devices XtraFix^®^, Large E.F.^®^, Dolphix^®^ and Hofmann III^®^.

The Citieffe’s External Fixator Dolphix^®^ showed the largest area free of distortions from the joint line in all the configurations (Figure 4).

## 4. Discussion

Davison et al. have shown some of the consequences that can be observed with MRI utilization on patients with external fixators. They reported that the Stryker Hoffman II Hybrid external fixator exhibited greater than 10kg of magnetic force 30 cms outside of the MRI portal entrance. The study further examined nine other external fixators with varying degrees of magnetic force [21]. At the time, none of the implants was an MRI-conditional instrumentation system of PEEK-CA30 and ERGAL 7075 aluminum alloy pin clamp like the Dolphix^®^, from Citieffe.

The amount of force reported by Davison et al. can cause pain to the patient at the site that is already severely injured and cause indirect movements of the bone fragments, as well as of the fixator’s components. Havsteen at al. have noted the problems surrounding motion artifacts in MRI use and stated that these artifacts could be minimized by keeping the patients comfortable [30]; however, with up to 10 kg of magnetic force on an injured extremity, it would be difficult to do so. Some authors may point to the availability of MRI-compatible implants. External fixator and MRI are two strategic tools in management of the trauma patient. The debate about the possibility to perform MRI in the presence of external fixator is more than 20 years old. After the safety concern has been largely investigated [1,7,8,14], the current discussion is about the efficacy of MRI in the presence of artifacts due to the presence of metallic component of external fixators. This study showed that, in the conditions described above, the MRI performed on instrumented legs provided images useful for more accurate diagnosis. The blinded radiologist asked to declare the quality of the images voted equally 5 out of 5 for all the images without detecting differences between the devices. The adopted scaling grade is not validated in literature, but it was previously used in different studies [27,31]. A similar comparison was made in 2015 by Elsissy et al., who investigated the distortion effect of two well-known brands of external fixators undergoing an MRI. Elsissy et al. concluded that even in presence of increased artifacts, all images were of diagnostic quality [20].

Our study compared whether any quantity difference exists in the quality of images between legs free of devices (control group) and legs that were instrumented. To answer the question, the SI, SNR and CNR were calculated on five different ROI in all the 20 configurations (four legs not instrumented and 16 instrumented with four different brands of external fixators). The statistical analysis showed there were significant differences between the control group and the groups of instrumented legs when considering only the SI in the ROIs’ bone marrow (*p* = 0.014) and patellar tendon (*p* = 0.012). However, the analysis of SNR and CNR in the same ROIs did not confirm the results (*p* = not significant). We can assume that the quantification of images showed no or minimal differences in quality of images performed with or without external fixators.

To evaluate if any difference existed between the brands of external fixators considered in the study, the same parameters were compared between legs instrumented with the different brands. Few statistically significant differences were detected among the devices. The ANOVA test run between the groups (excluding the control group) showed no significant differences in SI, SNR and CNR. We underline that the external fixator of Stryker was the only one that never showed differences with control group.

The method of comparing images by SNR and CNR is not completely validated in literature, but it is widely used. Kuhl et al. twice adopted the method in 2005 when comparing diagnostic ischemic lesions in diffusion weighted MRI compared to conventional phase and then differentiating 3.0 to 1.5 tesla MRI [27,31] The process was repeated by the previously mentioned study of Elsissy et al. elaborated on two brands of external fixators by SNR [20]. Morandi et al. in 2019 adopted this method in order to compare the stability of different configurations of external fixator around the knee. They showed that the “H” configuration is the more stable and less interfering with MRI [28,29]. The “H” configuration was utilized in this study.

The main novelty reported in this study consists in the finding that the different brand of external fixators was not differentiated by the quality of MRI images focusing on the ROIs, but by the artifacts of the images due to the presence of their pins. This was never considered in literature before. The reliability of MRI images of instrumented legs was previously discussed focusing on regions of interest far from the pins, as we did. However, the pins are mandatorily metallic (no other solutions exist in commerce) and obviously the distortion increases around those [32]. The last generation of external fixators allows physician to choose titanium pins in place of non-ferromagnetic stainless-steel pins, but the pins remain the main source of artifact in MRI. However, the length of the area where the distortion altered the images was never considered before. This was calculated in this study in a range between 53.17 ± 8.19 mm and 17 ± 5.4 mm per ExtraFix^®^, Zimmer and Dolphix^®^, Citieffe, the devices that showed the largest and shortest area of distortion, respectively. In the middle, Large External Fixator^®^ of DePuy Synthes and Hofmann III^®^ of Stryker showed diameters of the area of distortion of 45.07 ± 4.33 mm, 37.12 ± 10.17 mm, respectively. This led the authors to calculate the length of the area free of distortion from the joint in all legs, creating a means per brand. This showed a significant difference between the devices (*p* = 0.041). Dolphix^®^ of Citieffe was the external fixator that showed the largest area free of distortion in all the configurations with the overall clearest images.

The clinical application of the findings is considerable. It is our idea that the length of the area of distortion proper to the adopting device should be known by the physician. Several MRI findings could be clear by the presence of the artifacts. Taking for example the bone marrow edema, this is a quite common finding in knee trauma [33]. In proton density-weighted images (the same adopted in this study), this is described as ill-defined subchondral areas of high signal intensity, [33] and we showed this is the same distortion due to the presence of pins. Knowing the length of distortion due to pins could help in developing a diagnosis; thus, this should be considered when implanting the external fixator.

The limitations of this study are due to the small number of samples used. A larger number of tests should be done, especially focusing on the pins’ effect, and perhaps testing different materials. An in vivo investigation would help understanding the real importance of the finding and how much the artifacts of pins could affect the diagnosis.

## 5. Conclusions

MRI and external fixator are two strategic tools in the management of trauma patients. The concern of safety of performing MRI in the presence of the external fixators seems be solved, therefore, moving the debate towards the quality of images obtained in this condition. The main limit of the procedure is, in fact, still due to the presence of metallic components, as the pins can create artifacts for several centimeters. This aspect differentiated the devices and physicians should consider this when placing the pins. This study showed, once again, that MRI imaging performed in the presence of external fixators is possible and can provides images of excellent diagnostic quality, especially if the external fixators are manufactured with PEEK-CA30 and ERGAL 7075 like Dolphix^®^ and utilize titanium pins. 

## Data Availability

Not applicable.
